# Revision and postoperative complication rates of conversion total hip arthroplasty after cephalomedullary nailing of intertrochanteric femur fractures: a systematic review and meta-analysis

**DOI:** 10.1007/s00590-026-04681-6

**Published:** 2026-02-19

**Authors:** Harmon S. Khela, Harsh Wadhwa, Henry J. Wong, L. Henry Goodnough

**Affiliations:** 1 Perelman School of Medicine, University of Pennsylvania, Philadelphia, USA; 2 Department of Orthopaedic Surgery, Stanford Medicine, Stanford, USA

**Keywords:** Conversion total hip arthroplasty, Intertrochanteric fracture, Cephalomedullary nailing, Periprosthetic fracture, Revision, Complications

## Abstract

**Purpose:**

Management of failed intertrochanteric (IT) fracture fixation often requires conversion total hip arthroplasty (cTHA), but complication and reoperation rates remain poorly characterized in the literature. This study aimed to characterize revision and postoperative complication rates for cTHA after cephalomedullary nailing of IT fractures.

**Methods:**

A systematic review and meta-analysis was conducted following PRISMA guidelines. PubMed and Embase were queried for studies examining cTHA outcomes after prior cephalomedullary nailing for IT fractures. The primary outcome was revision rate, while secondary outcomes included rates of infection, dislocation, subsidence, and periprosthetic fracture (PPF). A random-effects model was employed to pool overall and implant-specific complication rates.

**Results:**

Seventeen studies with 1,253 cTHA patients met inclusion criteria. Implant type was specified in 799 cases: primary cementless (47.9%), primary cemented (38.8%), diaphyseal cementless (12.0%), and diaphyseal cemented (1.3%). Overall revision rate was 6.7%, infection rate was 3.3%, dislocation rate was 4.5%, and PPF rate was 5.9%. Subgroup analysis across six studies allowed for comparison between primary cementless (*n* = 265), primary cemented (*n* = 220), and diaphyseal cementless (*n* = 17) implants. Cemented stems had a revision rate of 2.4%, an infection rate of 1.8%, a dislocation rate of 2.4%, a subsidence rate of 3.8%, and a PPF rate of 3.2%, compared to cementless (10.1%, 2.8%, 3.3%, 8.1%, 7.7%) and diaphyseal cementless (7.0%, 8.9%, 18.3%, 7.0%, 7.0%) stems.

**Conclusion:**

cTHA is an effective salvage option for failed IT fracture fixation but carries notable postoperative risks compared to primary THA. Optimal outcomes require careful femoral component selection tailored to patient-specific factors.

**Supplementary Information:**

The online version contains supplementary material available at 10.1007/s00590-026-04681-6.

## Introduction

Intertrochanteric (IT) femur fractures are common injuries, especially among the elderly, accounting for 42.0% of all hip fractures [1]. The global incidence of hip fractures continues to rise as the population ages, with the total annual number of fractures projected to exceed 4.5 million by 2050 [2]. IT fractures are primarily managed with indirect reduction and internal fixation with cephalomedullary nails or dynamic hip screws. Despite advancements in fixation methods, fixation failure can occur due to mechanical complications like intramedullary nail cutout, delayed or incomplete fracture healing, postoperative infection, or the development of post-traumatic arthritis. Failure rates remain as high as 16%, particularly in osteoporotic patients [3].

The primary approaches for managing failed internal fixation of IT fractures are revision fixation or conversion to hemiarthroplasty or total hip arthroplasty. Treatment decision hinges on factors like bone quality, the failed fixation construct, and patient health conditions [4]. Among elderly patients, conversion total hip arthroplasty (cTHA) and hemiarthroplasty are widely accepted as effective salvage options when fixation devices fail [5]. cTHA is associated with extended operative time and increased blood loss relative to primary THA, due to the need for implant removal, revision strategies, and specialized components [6, 7]. cTHA for failed IT fracture fixation is also associated with prolonged hospital stays and higher costs compared to primary THA [6, 7]. Importantly, the timing and cause of failure often dictate the appropriate implant strategy. Tate et al. showed that early cTHA, defined as within 1 year of fracture fixation, was associated with significantly higher risks of reoperation, infection, periprosthetic fracture, dislocation, and medical complications compared to conversions performed after 1 year [8]. Grayson et al. corroborated these findings and found that patients undergoing early conversion within 6 months experienced nearly double the overall complication rate, including markedly higher rates of deep infection, revision surgery, and perioperative transfusion, compared to those converted after 6 months [9].

Despite the increasing use of conversion total hip arthroplasty (cTHA) as a salvage procedure after failed intertrochanteric fracture fixation, postoperative complication and revision rates remain variably reported, and implant-specific outcomes are poorly characterized. The primary aim was to estimate the pooled revision rate following cTHA after failed cephalomedullary nailing of intertrochanteric femur fractures. Secondary aims were to estimate pooled rates of postoperative infection, dislocation, periprosthetic fracture, and femoral stem subsidence. A secondary exploratory analysis was performed to descriptively summarize outcomes stratified by femoral component fixation strategy.

## Methods

### Search strategy

This systematic review and meta-analysis were performed in accordance with the PRISMA (Preferred Reporting Items for Systematic Reviews and Meta-Analyses) guidelines [10]. A comprehensive search of the PubMed and Embase databases was completed on May 31, 2024, to identify articles reporting on outcomes following cTHA for patients who failed prior IT fracture fixation using keywords and medical subject headings. A combination of search terms such as “fracture fixation,” “open reduction and internal fixation,” “intertrochanteric,” “failure, “postoperative complications,” and “hip arthroplasty,” were used to query each database. The full search strategy can be found in Supplemental Table 1.

### Inclusion and exclusion criteria

**Fig. 1:**
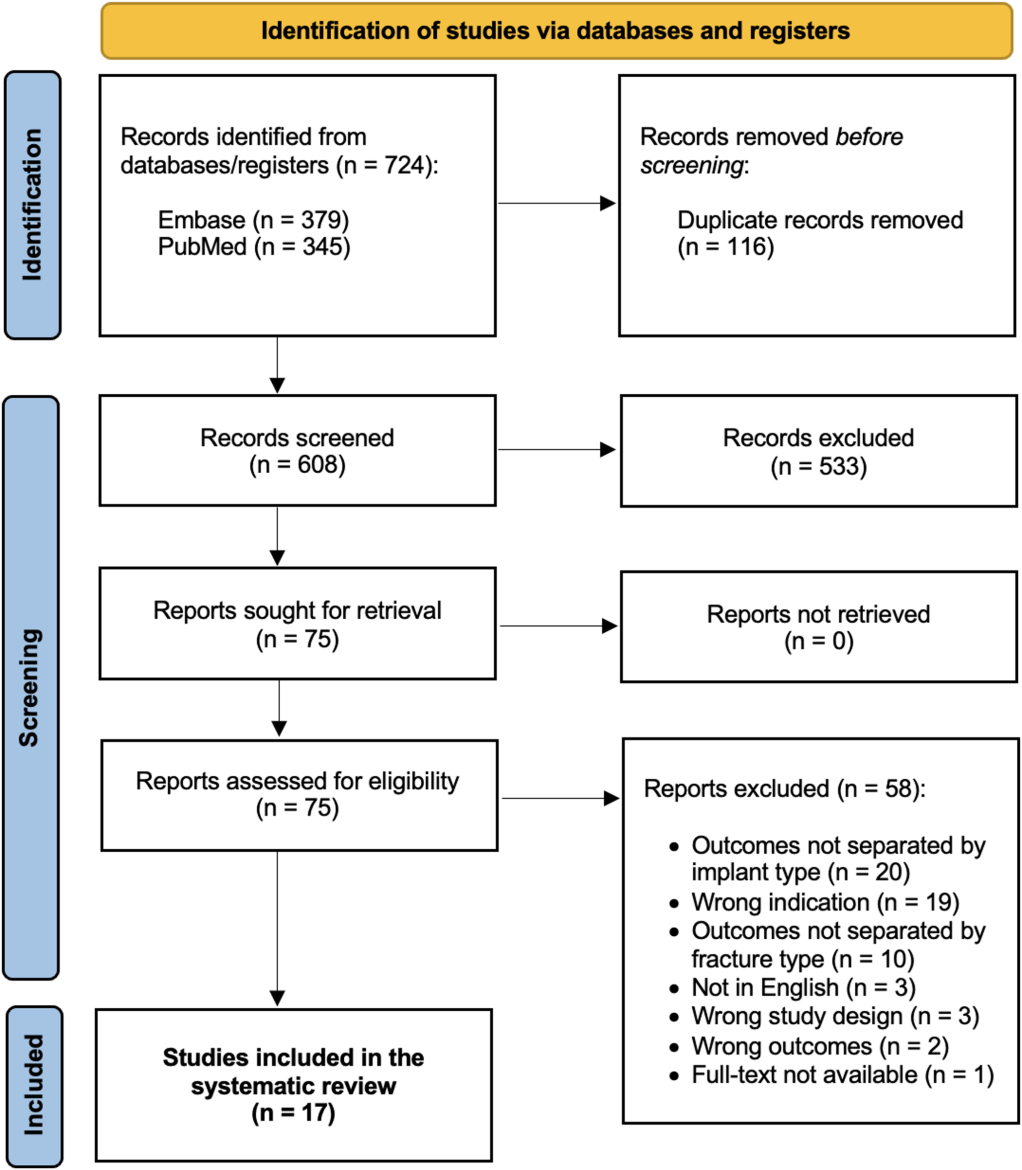
PRISMA (Preferred Reporting Items for Systematic Review and Meta-Analyses) flowchart describing the systematic review process

Search results were imported into Covidence (Melbourne, Australia), and duplicates were removed. Titles and abstracts were screened based on inclusion and exclusion criteria, followed by a full-text review to assess eligibility. Two authors (H.S.K. and H.W.) independently reviewed the studies, resolving any discrepancies through discussion to reach consensus. Studies were deemed eligible if they met the following inclusion criteria: (1) included a minimum of three patients who underwent cTHA for failed cephalomedullary nail fixation of IT fracture, (2) provided defined data for the patient population, (3) reported revision rate after cTHA, and (4) were published in English. Studies were included only if pertinent data on the patients who had prior cephalomedullary nail fixation could be separately extracted, even if the total cohort included patients who received other fixation types (plate, dynamic hip screw, etc.). Similarly, if a study included a cohort of patients with IT fractures and other fracture types (subtrochanteric, femoral neck, etc.) but did not stratify outcomes based on fracture type or fixation method, it was excluded. Studies were excluded if they (1) did not meet the inclusion criteria, (2) reported on patients with infection or tumor/pathologic fracture cases, or were (3) case reports, commentaries, systematic and literature reviews, or surgical technique articles, (4) not performed on human patients, or (5) not available in full text. Figure 1 provides an overview of the search strategy and screening process.

### Data collection

Two reviewers extracted data from each study using a standardized data form. The following data were extracted: authors and publication year, study design, sample size, follow-up duration, patient demographic characteristics, initial implant type, cTHA approach, implant used in cTHA, intraoperative details, and postoperative outcomes. Intraoperative data variables obtained were operative time, length of stay, estimated blood loss, and need for transfusion. Clinical outcomes and complications of interest were rates of revision, infection, dislocation, subsidence, PPF, and any other complications reported explicitly in the text. In studies where a fraction of the total cohort underwent cTHA, the patient demographics (sample size, mean age, and gender distribution) only for those who underwent THA was not typically available. Therefore, in these cases, the demographic data we extracted was for the entire sample. A subgroup analysis of studies that reported outcomes based on femoral component type was performed, though implant-specific stratification was inconsistently reported across the literature.

### Risk of bias assessment

The methodological quality and risk of bias for each included study were assessed using the Newcastle-Ottawa Scale (NOS) [11]. This tool evaluates non-randomized studies based on three primary domains: selection of study groups, comparability of groups, and ascertainment of either exposure or outcome, depending on the study design. Each study was rated on a scale of up to nine points. Scores of 7–9 indicated low risk of bias, 4–6 indicated high risk of bias, and 0 to 3 indicated very high risk of bias. While NOS scores were used to describe overall study quality, sensitivity analyses stratified by risk-of-bias category were not performed due to the limited number of studies.

### Data analysis

Statistical analyses were performed using R version 4.4.1 and the metafor package (version 4.60) [12]. To address rare outcomes, a logit transformation was applied with an adjustment of 0.5 for studies with zero counts in revision, infection, dislocation, and PPF rates. This continuity correction was applied to avoid exclusion of clinically relevant cohorts reporting no complications, although it may influence effect estimates for infrequent outcomes. Combined rates were estimated using random-effects models and displayed through forest plots to provide a visual summary of complications after cTHA across studies. Publication bias assessment was performed using funnel plots and small study effects were tested using Egger’s regression tests. Heterogeneity was evaluated using Cochran’s Q Test and quantified using Higgin’s I^2^ and τ^2^. All statistical tests used a two-sided significance level of ⍺ = 0.05.

## Results

The initial search yielded 724 studies, with 116 duplicate records removed before screening. After screening the titles and abstracts of the remaining 608 studies, 533 were excluded based on predefined inclusion and exclusion criteria, leaving 75 full-text articles for further assessment. Following full-text review, an additional 58 studies were excluded (Fig. 1). Ultimately, 17 studies met the eligibility criteria and were included in this systematic review [13–29]. Most of these were retrospective cohort or case series studies, with 13 Level III and 4 Level IV studies.

The risk of bias was relatively low, with an average NOS score of 8 (Table 1). Publication bias and small-study effects were assessed for pooled outcomes using funnel plot inspection and Egger’s regression test. Visual inspection of the funnel plots did not demonstrate clear asymmetry. Formal assessment using Egger’s regression did not indicate statistically significant asymmetry (*p* > 0.05) (Supplemental Table 2). There was no significant concern for heterogeneity for revision rate (I^2^ = 0.0%, τ^2^ = 0.000), infection rate (I^2^ = 23.2%, τ^2^ = 0.194), dislocation rate (I^2^ = 27.9%, τ^2^ = 0.171), and periprosthetic fracture rate (I^2^ = 6.4%, τ^2^ = 0.027). This was further confirmed using Cochran’s Q test (*p* > 0.05).

**Table 1:** Newcastle-Ottawa scale risk of bias assessment of included studies

Author (year)	Study design	Level of evidence	Selection				Comparability	Outcome			Quality
			Representativeness of exposed cohort (+)	Selection of nonexposed cohort (+)	Ascertainment of exposure (+)	Outcome not present before study (+)	Based on design and analysis (++)	Assessment of outcomes (+)	Follow-up length (+)	Adequacy of follow-up cohorts (+)	
Brunner (2016)	Retrospective Multicenter Cohort	Level III	+	+	+	+		+	+	+	7/9
Cardenas (2024)	Retrospective Multicenter Cohort	Level III	+	+	+	+	+	+	+	+	8/9
Gazzotti (2014)	Retrospective Case Series	Level IV	+	+	+	+		+	+	+	7/9
Godoy-Monzon (2020)	Retrospective Case Series	Level IV	+	+	+	+	+	+	+	+	8/9
Huang (2022)	Retrospective Cohort	Level III	+	+	+	+	+	+	+	+	8/9
Liu (2014)	Retrospective Case Series	Level IV		+	+	+		+	+	+	6/9
Mathur (2022)	Prospective Case Series	Level IV	+	+	+	+	+	+	+	+	8/9
Min (2019)	Prospective Cohort	Level III	+	+	+	+	++	+	+	+	9/9
Pui (2013)	Retrospective Multicenter Cohort	Level III	+	+	+	+	+	+	+	+	8/9
Schultz (2022)	Retrospective Cohort	Level III	+	+	+	+	++	+	+	+	9/9
Smith (2019)	Retrospective Cohort	Level III	+	+	+	+	+	+	+	+	8/9
Solarino (2013)	Retrospective Cohort	Level III	+	+	+	+		+	+	+	7/9
Soundarrajan (2024)	Retrospective Cohort	Level III	+	+	+	+		+	+	+	7/9
Yu (2020)	Retrospective Cohort	Level III	+	+	+	+	+	+	+	+	8/9
Yu (2016)	Retrospective Cohort	Level III	+	+	+	+	+	+	+	+	8/9
Zeng (2017)	Retrospective Cohort	Level III	+	+	+	+		+	+	+	7/9
Zhong (2021)	Retrospective Cohort	Level III	+	+	+	+		+	+	+	7/9

Across all studies, there were a total of 1253 cTHAs performed. Of these, cTHA implant type was specified in 799 cases. The most common implants used were primary cementless stems (47.9%), followed by primary cemented (38.8%), diaphyseal cementless (12.0%), and diaphyseal cemented (1.3%) stems. Among the studies that further specified implant details, the diaphyseal revision stems were modular taper-fluted designs (*n* = 28) or full-coated distally loading stems (*n* = 15). For primary cementless stems, reported designs included metaphyseal-fitting implants such as the Corail, CLS, and Taperloc stems. For primary cemented stems, the Exeter Universal stem—a polished, tapered cemented design—was the most commonly reported. Several studies, however, did not provide sufficient detail to determine specific stem type. The median follow-up time among the included studies was 27.5 months (range: 5.7–65.0 months). An overview of the demographics and intraoperative details for each study is outlined in Tables 2 and 3, respectively.

**Table 2:** Study characteristics and demographics

Author (year)	Study design	Sample size	Mean age (range or SD)	Females (%)	Mean follow-up, months (range or SD)	Initial implant	Approach	Number of cTHAs	Implant type in cTHA	Implant details
Brunner (2016)	Retrospective Multicenter Cohort	57	81 (8.9)	46 (80.7)	5.7	Proximal femoral nail antirotation (*n* = 47) Trochanteric fixation nail (*n* = 10)	–	27	–	–
Cardenas (2024)	Retrospective Multicenter Cohort	45	69.78 (12.14)	32 (71.1)	24	Intramedullary nail (*n* = 45)	–	45	–	–
Gazzotti (2014)	Retrospective Case Series	10	75.1 (12.67)	9 (90.0)	26.2 (11.93)	Gamma nail (*n* = 10)	–	10	Primary cementless (*n* = 8) Diaphyseal revision cementless (*n* = 2)	N/A
Godoy-Monzon (2020)	Retrospective Case Series	28	72.7 (10.5)	17 (60.7)	63.6 (60–84)	Cephalomedullary nail (*n* = 28)	Posterolateral	28	Diaphyseal revision cementless (*n* = 23) Diaphyseal revision cemented (*n* = 5)	MP Reconstruction Prosthesis (modular taper-fluted stem) (*n* = 28)
Huang (2022)	Retrospective Cohort	244	Uncemented THA: 68 (63–76) Cemented THA: 68 (64–77)	Uncemented THA: 60 (48.4) Cemented THA: 57 (47.5)	60 (50–67)	Proximal femoral nail antirotation (*n* = 244)	Lateral	244	Primary cementless (*n* = 124) Primary cemented (*n* = 120)	Taperloc stem (proximal metaphyseal-fitting, wedge-shaped, porous-coated cementless stem) (*n* = 124) Exeter Universal stem (polished, tapered cemented stem) (*n* = 120)
Liu (2014)	Retrospective Case Series	9	73.4 (63–82)	6 (85.7)	18 (12–24)	Proximal femoral nail antirotation (*n* = 7)	–	7	–	–
Mathur (2022)	Prospective Case Series	15	62.8 (9.1)	7 (46.7)	16.9 (7.9)	Proximal femoral nail antirotation (*n* = 15)	Posterolateral	15	Diaphyseal revision cementless (*n* = 15)	DePuy Solution System (extensively porous coated distally loading revision stem) (*n* = 15)
Min (2019)	Prospective Cohort	83	64.6 (19–87)	33 (39.8)	42 (15.6–108)	Cephalomedullary nail (*n* = 19)	Anterolateral	19	Primary cementless (*n* = 19)	Zimmer CLS stem (*n* = 19)
Pui (2013)	Retrospective Multicenter Cohort	31	65.1 (20.5)	19 (61.3)	35	Cephalomedullary nail (*n* = 31)	Posterolateral, Anterolateral	31	Primary cementless (*n* = 24) Primary cemented (*n* = 7)	N/A
Schultz (2022)	Retrospective Cohort	22	77.09 (8.83)	17 (77.3)	36.6 (28.4)	Cephalomedullary nail (*n* = 22)	Posterolateral, Anterolateral	22	Diaphyseal revision cementless (*n* = 17) Diaphyseal revision cemented (*n* = 5)	N/A
Smith (2019)	Retrospective Cohort	369	79.93 (5.86)	276 (74.8)	24	Cephalomedullary nail (*n* = 369)	–	369	–	–
Solarino (2013)	Retrospective Cohort	74	73.8 (7.85)	45 (60.8)	24	Cephalomedullary nail (*n* = 74)	Anterolateral	74	Diaphyseal revision cementless (*n* = 39) Primary cemented (*n* = 19) Primary cementless (*n* = 16)	N/A
Soundarrajan (2024)	Retrospective Cohort	39	61.5 (16.1)	20 (51.3)	27.5 (12–72)	Cephalomedullary nail (*n* = 16)	Anterolateral	16	Primary cementless (*n* = 16)	Zimmer Wagner SL stem (monoblock, tapered, distal-loading, grit-blasted titanium stem) (*n* = 16)
Yu (2020)	Retrospective Cohort	198	Uncemented THA: 66.84 (4.72) Cemented THA: 66.73 (5.12)	Uncemented THA: 53 (54.1) Cemented THA: 52 (52.0)	65 (60–69)	Proximal femoral nail antirotation (*n* = 198)	–	198	Primary cementless (*n* = 98) Primary cemented (*n* = 100)	Corail stem (proximal metaphyseal-fitting stem) (*n* = 98) Exeter Universal stem (polished, tapered cemented stem) (*n* = 100)
Yu (2016)	Retrospective Cohort	70	70.07 (11.76)	33 (47.1)	31.67 (5.24)	Proximal femoral nail antirotation (*n* = 70)	Posterolateral, Anterolateral	70	Primary cementless (*n* = 36) Primary cemented (*n* = 34)	N/A
Zeng (2017)	Retrospective Cohort	72	76.93 (9.25)	41 (56.9)	47.92 (4.31)	Proximal femoral nail antirotation (*n* = 72)	Posterolateral	72	Primary cementless (*n* = 42) Primary cemented (*n* = 30)	N/A
Zhong (2021)	Retrospective Cohort	18	67.3 (38–92)	8 (80.0)	24.7 (12–36)	Intramedullary nail (*n* = 6)	Posterolateral	6	–	–

*cTHA* conversion total hip arthroplasty, *SD* standard deviation

**Table 3:** Intraoperative details and postoperative outcomes

Author (year)	Number of cTHAs	Mean operative time, minutes (SD)	Mean LOS, days (SD)	Mean EBL, mL (SD)	Need for Transfusion, *n* (%)	Number of Transfused Units	Revision, *n* (%)	Infection, *n* (%)	Dislocation, *n* (%)	Subsidence, *n* (%)	PPF, *n* (%)	Other complications, *n*
Brunner (2016)	27	–	–	–	0 (0.0)	–	1 (3.7)	0 (0.0)	1 (3.7)	0 (0.0)	0 (0.0)	–
Cardenas (2024)	45	–	3.77 (2.58)	576.83 (366.59)	0 (0.0)	–	6 (13.3)	4 (8.9)	1 (2.2)	0 (0.0)	2 (4.4)	Falls (*n* = 2) Acute renal failure (*n* = 1) Heterotopic ossification (*n* = 1) IT band syndrome (*n* = 1) Myocardial infarction (*n* = 1) Potential metal allergy (*n* = 1)
Gazzotti (2014)	10	–	–	–	7 (70.0)	2, 3, or 4 units	1 (10.0)	0 (0.0)	2 (20.0)	0 (0.0)	0 (0.0)	Persistent thigh pain (*n* = 3) Ipometria > 2 cm (*n* = 1)
Godoy-Monzon (2020)	28	–	–	–	–	–	0 (0.0)	1 (3.6)	2 (7.2)	0 (0.0)	0 (0.0)	Sympotomatic abductor deficiency (*n* = 1)
Huang (2022)	244	–	–	–	–	–	11 (4.5)	7 (2.9)	4 (1.6)	11 (4.5)	10 (4.1)	Insufferable hip pain (*n* = 7) Lower limb shortening (*n* = 6) Thrombotic events (*n* = 2)
Liu (2014)	7	–	–	–	0 (0.0)	–	0 (0.0)	0 (0.0)	0 (0.0)	0 (0.0)	0 (0.0)	–
Mathur (2022)	15	–	–	461.3 (22.3)	15 (100.0%)	1 unit (*n* = 12), 2 units (*n* = 3)	0 (0.0)	1 (6.7)	1 (6.7)	0 (0.0)	0 (0.0)	Intraoperative hypotension (*n* = 1)
Min (2019)	19	–	–	–	–	–	3 (15.8)	0 (0.0)	1 (5.3)	0 (0.0)	1 (5.3)	–
Pui (2013)	31	–	–	–	–	–	0 (0.0)	1 (3.2)	2 (6.4)	1 (3.2)	3 (9.7)	Urinary tract infection (*n* = 1) Pulmonary embolism (*n* = 1) GI bleed (*n* = 1) Acute renal failure (*n* = 1) Abductor tendon deficiency (*n* = 1) Heterotopic ossification (*n* = 2) Intraoperative nerve injury (*n* = 1)
Schultz (2022)	22	176.3 (54.7)	5.5 (2.9)	600 (240)	–	–	3 (13.6)	2 (9.1)	2 (9.1)	0 (0.0)	0 (0.0)	Sepsis (*n* = 10) Acute kidney injury (*n* = 1) Urinary tract infection (*n* = 1) Anemia (*n* = 7)
Smith (2019)	369	–	5.3 (3.5)	–	–	–	24 (6.5)	3 (0.8)	9 (2.4)	0 (0.0)	0 (0.0)	Care involving other specified rehabilitation procedure leading to revision (*n* = 3) Urinary tract infection (*n* = 3) Other mechanical complication of orthopaedic device, implant or graft (*n* = 2) Myasthenia gravis (*n* = 1) Atrial flutter (*n* = 1) SA node dysfunction (*n* = 1) Acute venous thromboembolism (*n* = 1) Bacterial pneumonia (*n* = 1)
Solarino (2013)	74	117 (76–192)	–	585 (430–1720)	43 (58.1%)	3 or more units	4 (5.4)	3 (4.0)	5 (6.8)	0 (0.0)	8 (10.8)	Death due to pulmonary embolism (*n* = 2) Ischemic myocardial infarction (*n* = 1)
Soundarrajan (2024)	16	–	–	–	–	–	2 (12.5)	0 (0.0)	0 (0.0)	0 (0.0)	2 (12.5)	–
Yu (2020)	198	–	–	–	–	–	14 (7.1)	0 (0.0)	6 (3.0)	18 (9.1)	13 (6.6)	Unbearable hip pain (*n* = 7)
Yu (2016)	70	–	–	–	–	–	0 (0.0)	2 (2.9)	4 (5.7)	0 (0.0)	5 (7.1)	Urinary tract infection (*n* = 2) Pulmonary embolism (*n* = 2) Atrial fibrillation (*n* = 2) Acute renal failure (*n* = 1) Abductor tendon deficiency (*n* = 2) Heterotopic ossification (*n* = 4) Intraoperative nerve injury (*n* = 1)
Zeng (2017)	72	–	–	–	–	–	0 (0.0)	2 (2.8)	1 (1.4)	0 (0.0)	3 (4.2)	Urinary tract infection (*n* = 1) Atrial fibrillation (*n* = 2) Prosthetic instability (*n* = 1) Limb length discrepancy (*n* = 1) Abductor tendon deficiency (*n* = 2) Heterotopic ossification (*n* = 1) Intraoperative nerve injury (*n* = 1)
Zhong (2021)	6	–	–	327.5 (81.7)	–	–	0 (0.0)	0 (0.0)	1 (16.7)	0 (0.0)	0 (0.0)	–

*cTHA* conversion total hip arthroplasty, *EBL* estimated blood loss, *LOS* length of stay, *PPF* periprosthetic fracture, *SD* standard deviation

**Fig. 2:**
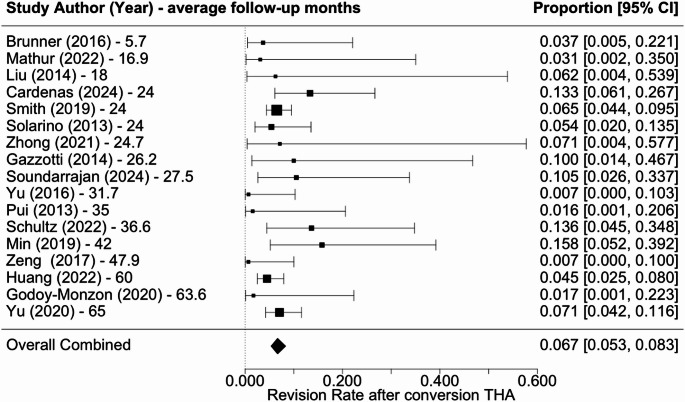
Forest plot of revision rate following cTHA

**Fig. 3:**
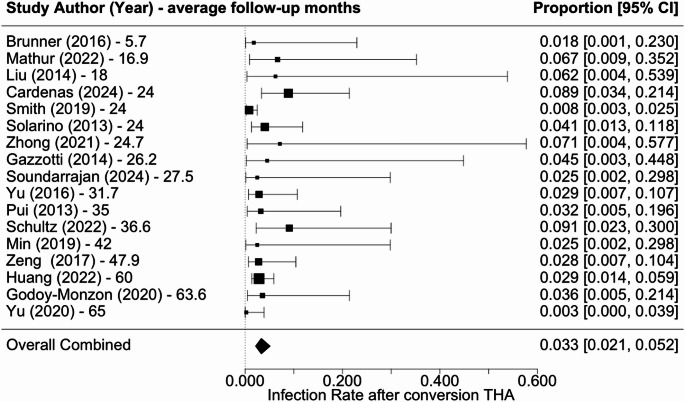
Forest plot of infection rate following cTHA

**Fig. 4:**
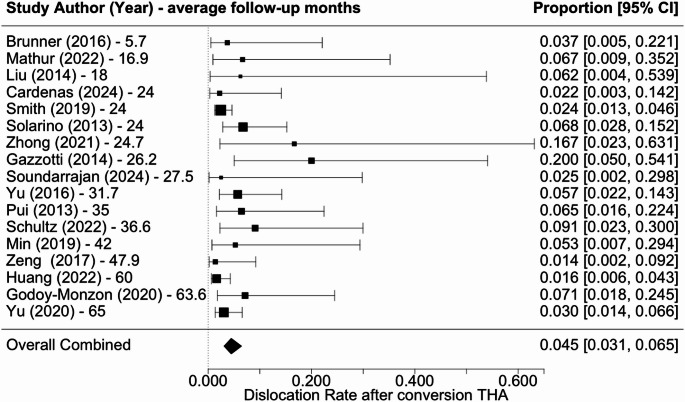
Forest plot of dislocation rate following cTHA

**Fig. 5:**
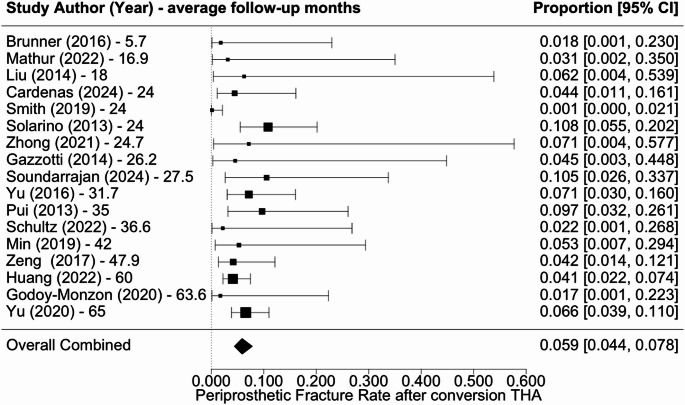
Forest plot of periprosthetic fracture rate following cTHA

Overall revision rate was 6.7% (5.3–8.3%) (Fig. 2), infection rate was 3.3% (2.1–5.2%) (Fig. 3), dislocation rate was 4.5% (3.1–6.5%) (Fig. 4), and PPF rate was 5.9% (4.4–7.8%) (Fig. 5). Subsidence was reported in only three studies at rates of 4.5%, 3.2%, and 9.1% [18, 22, 27]. Additional complications associated with cTHA were diverse, including frequent occurrences of urinary tract infections, heterotopic ossification, and abductor tendon deficiencies. Cardiovascular complications such as pulmonary embolism and atrial fibrillation, along with persistent pain issues like severe hip or thigh discomfort, were also documented.

An exploratory analysis was performed by implant type. There were six studies that stratified outcomes based on implant type: primary cementless (*n* = 265), primary cemented (*n* = 220), and diaphyseal cementless (*n* = 17) stems (Supplemental Table 3) [16, 18, 20, 21, 26, 27]. The overall pooled revision rate was 10.1% (7.0-14.5%) for cementless implants, 2.4% [1.0–5.6%] for cemented implants, and 7.0% (0.9–37.2%) for diaphyseal cementless implants. Infection rates were 2.8% (1.3–6.1%) for cementless, 1.8% (0.5–6.2%) for cemented, and 8.9% (1.8–34.7%) for diaphyseal cementless implants, while dislocation rates were 3.3% (1.6–6.6%), 2.4% (1.0-5.6%), and 18.3% (1.7–74.4%), respectively. Subsidence occurred in 8.1% (4.3–15.0%) of cementless implants, 3.8% (1.9–7.5%) of cemented implants, and 7.0% (0.9–37.2%) of diaphyseal cementless implants, while PPF was reported in 7.7% (4.7–12.5%), 3.2% (1.5–6.5%), and 7.0% (0.9–37.2%), respectively. However, it is important to note that stem choice may have been highly confounded by baseline patient factors and bone quality.

## Discussion

The management of failed IT fracture fixation remains a significant challenge despite advancements in fixation techniques. Existing literature on outcomes following cTHA for failed IT fracture fixation is limited. This systematic review and meta-analysis found a pooled revision rate of 6.7% after cTHA for failed cephalomedullary nailing of IT fractures. Additionally, we characterized rates of infection (3.3%), dislocation (4.5%), and PPF (5.9%). Subgroup analysis of femoral component type identified variation in revision and complication rates among primary cemented, primary cementless, and diaphyseal cementless implants.

Compared to primary THA, current evidence has largely supported that cTHA carries a higher complication burden [7, 30–33]. Our study found a revision rate of 6.7% following cTHA for failed cephalomedullary nailing of IT fractures, aligning with prior research. In a national database study, the authors matched primary THA and cTHA patients based on age, gender, and other comorbidities and reported a 4.0% revision rate at 90 days and 10.1% at one year for cTHA, which was notably elevated compared to 1.5% and 2.8% for primary THA, respectively [30]. Similarly, in a single-center prospective database study composed of 163 matched primary and cTHA patients, revision surgery was more prevalent in the cTHA cohort (11.7%) than the primary THA cohort (4.9%) [7]. Schwarzkopf et al. identified and matched 119 cTHAs and 251 primary THAs and determined that patients undergoing cTHA had a greater likelihood of requiring diaphyseal fixation and revision type implant components in addition to having a longer hospital length of stay and longer operative time [31]. Ryan et al. also found that intraoperative complications in cTHA were four times higher than those seen in primary THA, with conversion cases requiring significantly more blood transfusions and experiencing a greater incidence of fractures [7]. Other studies have confirmed this trend of increased intraoperative and short-term postoperative medical complications in cTHA compared to primary THA [32, 33]. These findings, in conjunction with our results, emphasize that while cTHA is an important salvage option for failed IT fracture fixation, it is a higher risk procedure than primary THA.

Moreover, in our study, rates of infection (3.3%), dislocation (4.5%), and periprosthetic fractures (5.9%) following cTHA similarly surpassed the rates typically reported for primary THA. Douglas et al. noted that cTHA is associated with higher rates of periprosthetic joint infection (7.7% vs. 1.4% at 90 days; 11.2% vs. 2.3% at 1 year; 12.2% vs. 2.4% at 2 years), dislocations (4.5% vs. 2.0% at 90 days), and mechanical complications (5.5% vs. 1.0% at 90 days) compared to primary THA [34]. In a different study, dislocation rates in cTHA (8.0%) were greater than primary THA (2.5%), although PPF rates were similar (3.1% vs. 3.7%) [26]. Other comparisons between cTHA and primary THA have indicated that cTHA is consistently associated with an elevated rate of postoperative surgical site infections [24]. Such evidence reinforces our results and the observed trend that conversion procedures inherently entail greater surgical complexity and postoperative risk.

In this study, due to limited reporting detail, no formal between-group statistical comparisons could be performed based on implant type, even though implant-specific descriptive pooled estimates are presented. Nevertheless, there is a lack of agreement in existing literature on the optimal conversion method after failed IT fracture fixation. A recent meta-analysis by Dix et al. compared various conversion methods (e.g., cemented, uncemented, and hemiarthroplasty) after failed IT fracture fixation and found no substantial difference in complication rates across methods, suggesting that outcomes may be more dependent on underlying patient factors and the technical challenges inherent to conversion procedures [33]. Similarly, Luthringer et al. reported that while cTHA might offer slightly better functional outcomes compared to hemiarthroplasty, complication rates remained comparable [35]. However, specific studies have highlighted that implant type may influence outcomes, suggesting that factors beyond the arthroplasty type, such as fixation method and implant model, may play a pivotal role in determining complication rates and functional outcomes [28, 36]. For instance, Zeng et al. reported variable outcomes based on femoral stem selection in cTHA after IT fracture failure [28]. The authors highlighted that cemented stems generally had fewer revisions, while cementless and diaphyseal stems were associated with increased risks of subsidence and instability. Conversely, Thalody et al. did not find substantial differences in outcomes between primary cementless metaphyseal-engaging femoral stems and revision diaphyseal-engaging stems used in cTHA [36]. Many of these studies are likely limited by their sample size and long-term follow-up, limiting conclusions about the true effect size of femoral component type on patient outcomes.

Beyond differences in implant selection, several mechanical and anatomical factors intrinsic to conversion total hip arthroplasty may contribute to the elevated complication rates observed in this population. Prior fracture fixation and surgical exposure can compromise the abductor mechanism, increasing the risk of postoperative instability and dislocation. Additionally, altered proximal femoral anatomy, residual bone defects following implant removal, and stress risers from prior screw or nail tracks may predispose patients to periprosthetic fracture and stem subsidence. Technical challenges associated with removal of well-fixed cephalomedullary nails, including cortical damage during extraction, may further exacerbate bone loss and complicate femoral reconstruction. These factors likely interact with patient-specific characteristics such as bone quality and fracture pattern, the importance of selecting femoral components based on patient-specific anatomical and clinical factors to mitigate complication risks and improve outcomes.

There are several limitations that should be acknowledged. First, our analysis was limited to studies retrieved from two databases (PubMed and Embase). The included studies were largely of Level III and IV evidence, which inherently limits the reliability of our conclusions and introduces potential bias. Our attempt to compare complication rates by implant type was constrained by the limited number of studies providing granular stratified data, thereby limiting any conclusions from our findings on implant-related outcomes. Additionally, our analysis may be subject to selection bias, as patients who underwent cTHA with cemented stems were likely older, frailer, and had poorer bone quality, which may have influenced implant selection and the likelihood of being included in studies reporting revision outcomes. As such, cemented stems may have been preferentially used in patients with lower functional demands or higher medical risk, potentially skewing complication rates.

This study also focused on cTHA revision and complication rates without incorporating functional outcome measures such as the Harris Hip Score, which limits our ability to evaluate patient-centered functional results comprehensively. Such outcomes were also not reliably reported in the included studies. Moreover, there were inconsistencies in follow-up durations across studies, which may impact the reported complication rates; outcomes like revision, infection, or dislocation are influenced by the length of follow-up, and varying durations make direct comparisons challenging. This approach was necessary due to inconsistent reporting across studies and is acknowledged as a methodological limitation. In cases when THA-specific demographic data were unavailable, our use of cohort-level demographic data may have introduced misclassification bias. Although we used statistical methods to minimize bias in the meta-analysis, the innate variability in study design, patient demographics, and reporting practices introduces an element of heterogeneity that cannot be fully controlled. Future research with higher-level evidence, larger sample sizes, standardized outcome measures, and extended follow-up periods is necessary to address these gaps and provide more definitive guidance on cTHA after cephalomedullary nailing of IT fractures.

## Conclusion

cTHA is a viable option for failed cephalomedullary nail fixation of IT fracture, with a revision rate of 6.7%, and infection, dislocation, and PPF rates of 3.3%, 4.5%, and 5.9%. Cemented femoral components demonstrated the lowest revision (2.4%), infection (1.8%), dislocation (2.4%), subsidence (3.8%), and PPF (3.2%) rates compared to cementless and diaphyseal cementless stems, which carry different respective complication profiles. Although implant-specific trends were observed, the limited data preclude firm conclusions. Future studies with larger, stratified cohorts are needed to better evaluate the effect of femoral component type on outcomes. Ultimately, implant decisions should be tailored to the individual patient, weighing patient risk factors and functional demands.

## Supplementary Information

Below is the link to the electronic supplementary material.

Supplementary Material 1

Supplementary Material 2

Supplementary Material 3

## Data Availability

No datasets were generated or analysed during the current study.

## References

[1] Bhandari M, Swiontkowski M (2017) Management of acute hip fracture. N Engl J Med 377(21):2053–2062. 10.1056/NEJMcp1611090 29166235 10.1056/NEJMcp1611090

[2] Veronese N, Maggi S (2018) Epidemiology and social costs of hip fracture. Injury 49(8):1458–1460. 10.1016/j.injury.2018.04.015 29699731 10.1016/j.injury.2018.04.015

[3] Wu LF, Zhang TS, Li J, Huang H, Zhou CH, Li X (2024) Construction and validation of a nomogram prediction model for internal fixation failure after proximal femoral anti-rotation intramedullary nailing in the treatment of intertrochanteric fractures of the femur. Med (Baltim) 103(47):e40575. 10.1097/MD.0000000000040575 10.1097/MD.0000000000040575 PMC11596523 39809193

[4] Liu P, Jin D, Zhang C, Gao Y (2020) Revision surgery due to failed internal fixation of intertrochanteric femoral fracture: current state-of-the-art. BMC Musculoskelet Disord 21(1):573 Published 2020 Aug 22. 10.1186/s12891-020-03593-8 32828132 10.1186/s12891-020-03593-8 PMC7443291

[5] O’Connor CM, Young JR, Villacres Mori B et al (2023) Conversion total hip arthroplasty following extracapsular hip fracture fixation with a cephalomedullary device: a comprehensive review. Arch Orthop Trauma Surg 143(6):3525–3533. 10.1007/s00402-022-04570-7 35986745 10.1007/s00402-022-04570-7

[6] Ryan SP, DiLallo M, Attarian DE, Jiranek WA, Seyler TM (2018) Conversion vs primary total hip arthroplasty: increased cost of care and perioperative complications. J Arthroplasty 33(8):2405–2411. 10.1016/j.arth.2018.03.006 29656967 10.1016/j.arth.2018.03.006

[7] Tate JP, Reinhart NM, Bridges CA, Brown NM, Sherman WF (2025) Comparative outcomes of early versus late conversion to total hip arthroplasty following hip fracture fixation. J Arthroplasty Published Online January 21. 10.1016/j.arth.2025.01.015 10.1016/j.arth.2025.01.015 39848449

[8] Grayson W, Eikani C, Benson M, Jozefowski N, Brown NM (2024) High rate of complications with early conversion hip arthroplasty following fracture treatment. J Am Acad Orthop Surg Glob Res Rev 8(11) e24.00318. Published 2024 Nov 19. 10.5435/JAAOSGlobal-D-24-00318 10.5435/JAAOSGlobal-D-24-00318 PMC11578206 39792608

[9] Leiss F, Goetz JS, Schindler M et al (2024) Influence of bone mineral density on femoral stem subsidence after cementless THA. Arch Orthop Trauma Surg 144(1):451–458. 10.1007/s00402-023-05006-6 37578658 10.1007/s00402-023-05006-6

[10] Moher D, Liberati A, Tetzlaff J, Altman DG, PRISMA Group (2009) Preferred reporting items for systematic reviews and meta-analyses: the PRISMA statement. PLoS Med 6(7):e1000097. 10.1371/journal.pmed.1000097 19621072 10.1371/journal.pmed.1000097 PMC2707599

[11] Wells G, Shea B, O’Connell D et al (2000) The newcastle–ottawa scale (NOS) for assessing the quality of non-randomized studies in meta-analysis. Ott Hosp. ;3–4

[12] Balduzzi S, Rücker G, Schwarzer G (2019) How to perform a meta-analysis with R: a practical tutorial. Evid Based Ment Health 22(4):153–160. 10.1136/ebmental-2019-300117 31563865 10.1136/ebmental-2019-300117 PMC10231495

[13] Brunner A, Büttler M, Lehmann U et al (2016) What is the optimal salvage procedure for cut-out after surgical fixation of trochanteric fractures with the PFNA or TFN? A multicentre study. Injury 47(2):432–438. 10.1016/j.injury.2015.11.027 26748415 10.1016/j.injury.2015.11.027

[14] Cardenas J, Pfeil AN, Fertitta DK et al (2024) Orthopedic hardware type impacts case complexity in conversion total hip arthroplasty surgery. Arthroplast Today 26:101317 Published 2024 Feb 20. 10.1016/j.artd.2024.101317 38415066 10.1016/j.artd.2024.101317 PMC10897850

[15] Gazzotti G, Matino G, Tsatsis C, Sacchetti G, Baudi P, Catani F (2014) Causes and treatments of lag screw’s cut out after intramedullary nailing osteosinthesis for trochanteric fractures. Acta Biomed 85(2):135–143 25245649

[16] Godoy-Monzon D, Diaz Dilernia F, Piccaluga F, Cid Casteulani A, Turus L, Buttaro M (2020) Conversion total hip arthroplasty with a proximally modular, distal fixation reconstruction prosthesis following cephalomedullar nail failure. Hip Int 30(1suppl):26–33. 10.1177/1120700020937952 32907420 10.1177/1120700020937952

[17] Huang T, Zhang S, Liu X et al (2022) Mid-Term outcomes of cemented or uncemented total hip arthroplasty for failed proximal femoral nail antirotation following intertrochanteric femur fractures: A retrospective observational study. Geriatr Orthop Surg Rehabil 13:21514593221132400. 10.1177/21514593221132400 36238963 10.1177/21514593221132400 PMC9551333

[18] Liu JJ, Shan LC, Deng BY, Wang JG, Zhu W, Cai ZD (2014) Reason and treatment of failure of proximal femoral nail antirotation internal fixation for femoral intertrochanteric fractures of senile patients. Genet Mol Res 13(3):5949–5956. 10.4238/2014.August.7.10 25117353 10.4238/2014.August.7.10

[19] Mathur HH, Shah HS, Vishwanathan K (2022) Functional outcome of conversion total hip arthroplasty (CTHA) using uncemented distally loading femoral stem for failed fixation of proximal femoral nail - A case series. J Orthop 34:14–20. 10.1016/j.jor.2022.07.014 35992612 10.1016/j.jor.2022.07.014 PMC9382133

[20] Min BW, Lee KJ, Oh JK, Cho CH, Cho JW, Kim BS (2019) The treatment strategies for failed fixation of intertrochanteric fractures. Injury 50(7):1339–1346. 10.1016/j.injury.2019.05.012 31151758 10.1016/j.injury.2019.05.012

[21] Pui CM, Bostrom MP, Westrich GH et al (2013) Increased complication rate following conversion total hip arthroplasty after cephalomedullary fixation for intertrochanteric hip fractures: a multi-center study. J Arthroplasty 28(8 Suppl):45–47. 10.1016/j.arth.2013.04.048 23891060 10.1016/j.arth.2013.04.048

[22] Schultz BJ, Sicat C, Penev A, Schwarzkopf R, Egol KA (2022) Conversion total hip arthroplasty for early failure following unstable intertrochanteric hip fracture: what can patients expect? Arch Orthop Trauma Surg 142(12):3737–3745. 10.1007/s00402-021-04215-1 34657163 10.1007/s00402-021-04215-1

[23] Smith A, Denehy K, Ong KL, Lau E, Hagan D, Malkani A (2019) Total hip arthroplasty following failed intertrochanteric hip fracture fixation treated with a cephalomedullary nail. Bone Joint J 91–96. 10.1302/0301-620X.101B6.BJJ-2018-1375.R1. 101-B(6_Supple_B 10.1302/0301-620X.101B6.BJJ-2018-1375.R1 31146562

[24] Solarino G, Bizzoca D, Dramisino P et al (2023) Total hip arthroplasty following the failure of intertrochanteric nailing: first implant or salvage surgery? World J Orthop 14(10):763–770. 10.5312/wjo.v14.i10.763 37970621 10.5312/wjo.v14.i10.763 PMC10642404

[25] Soundarrajan D, Fanta HT, Singh R, Dhanasekararaja P, Rajkumar N, Rajasekaran S (2024) Outcomes of conversion total hip arthroplasty for failed fixation of intertrochanteric fractures with monoblock distal-loading reconstruction stem. Eur J Orthop Surg Traumatol 34(4):2113–2120. 10.1007/s00590-024-03907-9 38548874 10.1007/s00590-024-03907-9

[26] Yu W, Han X, Chen W et al (2020) Conversion from a failed proximal femoral nail anti-rotation to a cemented or uncemented total hip arthroplasty device: a retrospective review of 198 hips with previous intertrochanteric femur fractures. BMC Musculoskelet Disord 21(1):791 Published 2020 Nov 30. 10.1186/s12891-020-03806-0 33256693 10.1186/s12891-020-03806-0 PMC7702693

[27] Yu W, Zhang X, Zhu X (2016) Noteworthy complication rate following conversion total hip arthroplasty after the fixation of proximal femoral nail antirotation-Asia for intertrochanteric femur fractures. Int J Clin Exp Med 9(3):6825–6830

[28] Zeng X, Zhan K, Zhang L et al (2017) Conversion to total hip arthroplasty after failed proximal femoral nail antirotations or dynamic hip screw fixations for stable intertrochanteric femur fractures: a retrospective study with a minimum follow-up of 3 years. BMC Musculoskelet Disord 18(1):38. 10.1186/s12891-017-1415-6 28122548 10.1186/s12891-017-1415-6 PMC5264307

[29] Zhong G, Teng L, Li HB, Huang FG, Xiang Z, Cen SQ (2021) Surgical treatment of internal fixation failure of femoral peritrochanteric fracture. Orthop Surg 13(6):1739–1747. 10.1111/os.13110 34142451 10.1111/os.13110 PMC8523769

[30] Schwarzkopf R, Chin G, Kim K, Murphy D, Chen AF (2017) Do conversion total hip arthroplasty yield comparable results to primary total hip arthroplasty? J Arthroplasty 32(3):862–871. 10.1016/j.arth.2016.08.036 27687806 10.1016/j.arth.2016.08.036

[31] Lee YK, Kim JT, Alkitaini AA, Kim KC, Ha YC, Koo KH (2017) Conversion hip arthroplasty in failed fixation of intertrochanteric fracture: A propensity score matching study. J Arthroplasty 32(5):1593–1598. 10.1016/j.arth.2016.12.018 28089470 10.1016/j.arth.2016.12.018

[32] Baghoolizadeh M, Schwarzkopf R, The Lawrence D (2016) Dorr surgical techniques & technologies award: conversion total hip arthroplasty: is it a primary or revision hip arthroplasty. J Arthroplasty 31(9 Suppl):16–21. 10.1016/j.arth.2015.06.024 26160646 10.1016/j.arth.2015.06.024

[33] Dix DB, Araoye IB, Staggers JR et al (2019) A systematic review and meta-analysis of complications in conversion arthroplasty methods for failed intertrochanteric fracture fixation. J Clin Orthop Trauma 10(2):282–285. 10.1016/j.jcot.2018.02.007 30828194 10.1016/j.jcot.2018.02.007 PMC6383065

[34] Douglas SJ, Remily EA, Sax OC, Pervaiz SS, Delanois RE, Johnson AJ (2021) How does conversion total hip arthroplasty compare to primary? J Arthroplasty 36(7S):S155–S159. 10.1016/j.arth.2020.12.023 33422393 10.1016/j.arth.2020.12.023

[35] Luthringer TA, Elbuluk AM, Behery OA, Cizmic Z, Deshmukh AJ (2018) Salvage of failed internal fixation of intertrochanteric hip fractures: clinical and functional outcomes of total hip arthroplasty versus hemiarthroplasty. Arthroplast Today 4(3):383–391 Published 2018 Jul 10. 10.1016/j.artd.2018.06.002 30186926 10.1016/j.artd.2018.06.002 PMC6123233

[36] Thalody HS, Post ZD, Lutz RW, Czymek M, Ong AC, Ponzio DY (2024) Primary cementless femoral stems in conversion hip arthroplasty after failed fixation of intertrochanteric fractures. Orthopedics 47(1):e6–e12. 10.3928/01477447-20230517-04 37216568 10.3928/01477447-20230517-04

