# Supplementary material for: Revision and postoperative complication rates of conversion total hip arthroplasty after cephalomedullary nailing of intertrochanteric femur fractures: a systematic review and meta-analysis
Source: Eur J Orthop Surg Traumatol. 2026 Feb 19;36(1):107. doi: 10.1007/s00590-026-04681-6 (PMC12920368; doi:10.1007/s00590-026-04681-6)
Supplement: Supplementary file 1 — Supplementary Material 1 [file 590_2026_4681_MOESM1_ESM.docx]

**Supplemental Table 1.** Database search paramaters

| **Database** | **Search Paramaters** |
| --- | --- |
| PubMed | ((("Open reduction" AND "internal fixation") OR nail* OR "Fracture Fixation, Internal"[mesh] OR "Hip fractures"[mesh]) AND (failure OR failed OR "reoperation"[mesh] OR reoperat* OR salvage OR "Postoperative complications"[mh]) AND (intertrochanteric OR extracapsular OR intramedullary) AND ("Total Hip" OR "Arthroplasty, Replacement, Hip"[MeSH])) OR (nail* AND ("conversion total hip arthroplasty" OR CTHA)) |
| Embase | ('open reduction' AND 'internal fixation' OR nail* OR 'internal fracture fixation' OR 'hip fractures') AND (failure OR failed OR 'reoperation' OR reoperat* OR salvage OR 'postoperative complications') AND (intertrochanteric OR extracapsular OR intramedullary) AND ('total hip' OR 'hip arthroplasty'/exp OR 'hip arthroplasty') OR ((nail* OR intramedullary) AND ('conversion total hip arthroplasty' OR ctha)) |
