# Supplementary material for: Revision and postoperative complication rates of conversion total hip arthroplasty after cephalomedullary nailing of intertrochanteric femur fractures: a systematic review and meta-analysis
Source: Eur J Orthop Surg Traumatol. 2026 Feb 19;36(1):107. doi: 10.1007/s00590-026-04681-6 (PMC12920368; doi:10.1007/s00590-026-04681-6)
Supplement: Supplementary file 2 — Supplementary Material 2 [file 590_2026_4681_MOESM2_ESM.docx]

**Supplemental Table 2**. Heterogeneity was assessed using Higgin’s I^2^, τ^2^, Cochran’s Q Test. Publication bias assessment from small study effects was evaluated using Egger’s Test.

|  | Heterogeneity | | | | Publication Bias / Small-study Effects | |
| --- | --- | --- | --- | --- | --- | --- |
|  | I^2 | tau^2 | Cochran's Q Statistic | Cochran's Q P-value | Egger's Test Z-statistic | Egger's Test P-value |
| Revision Rate | 0.0% | 0.000 | 17.847 | 0.333 | -1.326 | 0.185 |
| Infection Rate | 23.2% | 0.194 | 17.300 | 0.367 | -0.259 | 0.795 |
| Dislocation Rate | 27.9% | 0.171 | 19.851 | 0.227 | 1.172 | 0.241 |
| Periprosthetic Fracture Rate | 6.4% | 0.027 | 16.611 | 0.411 | -1.738 | 0.082 |
