# Supplementary material for: Revision and postoperative complication rates of conversion total hip arthroplasty after cephalomedullary nailing of intertrochanteric femur fractures: a systematic review and meta-analysis
Source: Eur J Orthop Surg Traumatol. 2026 Feb 19;36(1):107. doi: 10.1007/s00590-026-04681-6 (PMC12920368; doi:10.1007/s00590-026-04681-6)
Supplement: Supplementary file 3 — Supplementary Material 3 [file 590_2026_4681_MOESM3_ESM.docx]

|  | **Primary Cementless** | | | | | | **Primary Cemented** | | | | | | **Diaphyseal Revision Cementless** | | | | | |
| --- | --- | --- | --- | --- | --- | --- | --- | --- | --- | --- | --- | --- | --- | --- | --- | --- | --- | --- |
| **Author (Year)** | Evaluable Patients | Revision | Infection | Dislocation | Subsidence | PPF | Evaluable Patients | Revision | Infection | Dislocation | Subsidence | PPF | Evaluable Patients | Revision | Infection | Dislocation | Subsidence | PPF |
| Gazzotti (2014) | 8 | 1 | 0 | 1 | 0 | 0 | — | — | — | — | — | — | 2 | 0 | 0 | 1 | 0 | 0 |
| Huang (2022) | 124 | 9 | 4 | 2 | 8 | 6 | 120 | 2 | 3 | 2 | 3 | 4 | — | — | — | — | — | — |
| Mathur (2022) | — | — | — | — | — | — | — | — | — | — | — | — | 15 | 0 | 1 | 1 | 0 | 0 |
| Min (2019) | 19 | 3 | 0 | 1 | 0 | 1 | — | — | — | — | — | — | — | — | — | — | — | — |
| Soundarrajan (2024) | 16 | 2 | 0 | 0 | 0 | 2 | — | — | — | — | — | — | — | — | — | — | — | — |
| Yu (2020) | 98 | 11 | 0 | 3 | 13 | 10 | 100 | 3 | 0 | 3 | 5 | 3 | — | — | — | — | — | — |
| Total | 265 | 26 | 4 | 7 | 21 | 19 | 220 | 5 | 3 | 5 | 8 | 7 | 17 | 0 | 1 | 2 | 0 | 0 |
|  | Overall Combined Rates | | | | | | | | | | | | | | | | | |
| Revision Rate | 0.101 [0.070, 0.145] | | | | | | 0.024 [0.010, 0.056] | | | | | | 0.070 [0.009, 0.372] | | | | | |
| Infection Rate | 0.028 [0.013, 0.061] | | | | | | 0.018 [0.005, 0.062] | | | | | | 0.089 [0.018, 0.347] | | | | | |
| Dislocation Rate | 0.033 [0.016, 0.066] | | | | | | 0.024 [0.010, 0.056] | | | | | | 0.183 [0.017, 0.744] | | | | | |
| Subsidence Rate | 0.081 [0.043, 0.150] | | | | | | 0.038 [0.019, 0.075] | | | | | | 0.070 [0.009, 0.372] | | | | | |
| PPF Rate | 0.077 [0.047, 0.125] | | | | | | 0.032 [0.015, 0.065] | | | | | | 0.070 [0.009, 0.372] | | | | | |

**Supplemental Table 3.** Postoperative outcomes stratified by implant type

PPF, periprosthetic fracture
